# Adult Ileo-Ileal Intussusception Secondary to a 9 cm Pedunculated Submucosal Lipoma: Clinical, Radiologic, and Pathologic Correlation

**DOI:** 10.7759/cureus.107353

**Published:** 2026-04-19

**Authors:** Luciano Ferrando, Raquel León Mirada, Johanna B Palacios Ball, José F Reoyo Pascual, Miguel A Álvarez Rico

**Affiliations:** 1 General Surgery, Hospital Universitario de Burgos, Burgos, ESP; 2 Pathology, Hospital Universitario de Burgos, Burgos, ESP

**Keywords:** adult intussusception, computed tomography, ileo-ileal intussusception, small bowel lipoma, small bowel obstruction, surgical resection

## Abstract

Adult intussusception is a rare clinical entity and is typically associated with an underlying structural lesion, in contrast to pediatric cases where idiopathic causes predominate. We report a case of ileo-ileal intussusception in a 21-year-old male secondary to a large pedunculated submucosal lipoma. The patient presented with recurrent episodes of abdominal pain initially attributed to ileitis. Sequential computed tomography (CT) scans demonstrated a persistent ileo-ileal intussusception and identified a fat-density intramural lesion, allowing preoperative suspicion of a submucosal lipoma as the lead point. Urgent exploratory laparotomy revealed a long-segment ileo-ileal intussusception caused by a large pedunculated intraluminal mass. Manual reduction was performed, followed by segmental small-bowel resection and a stapled anastomosis. Histopathologic examination confirmed a 9 cm submucosal lipoma composed of mature adipose tissue, without evidence of ischemia or malignancy. The postoperative course was uneventful. This case highlights the diagnostic value of CT imaging in identifying fat-containing lead points and illustrates the radiologic, surgical, and pathologic correlation characteristic of lipoma-induced intussusception in young adults.

## Introduction

Intussusception in adults is rare, accounting for approximately 1-5% of all cases and about 1% of bowel obstructions [1,2]. Computed tomography (CT) is the imaging modality of choice in adult intussusception and can demonstrate characteristic findings such as the "target" or "sausage" sign [3].

Since the first systematic descriptions of adult intussusception in the mid-20th century, the condition has been recognized as a rare entity typically requiring surgical management. While exact incidence figures remain difficult to establish due to the rarity of reported cases, large institutional series suggest that lipomas account for only a small fraction of identifiable lead points in adult intussusception, with fewer than 50 cases of small bowel lipoma-induced intussusception reported in the English-language literature. Prognosis is largely determined by the underlying etiology, with benign lead points such as lipomas carrying an excellent outcome following surgical resection, in contrast to malignant causes, which are associated with significantly worse long-term prognosis.

Adult cases are commonly associated with an identifiable pathological lead point, frequently neoplastic [4]. Large series describe the clinical spectrum of adult intussusception and support surgical management given the high likelihood of underlying pathology [5]. More recent systematic reviews and meta-analyses have further confirmed its rarity and the importance of surgical management in the vast majority of cases [6].

Small bowel lipomas are uncommon benign tumors composed of mature adipose tissue; ultrasound and CT may aid characterization in appropriate clinical contexts [7]. Retrospective reviews further reinforce that adult intussusception often requires surgical exploration and treatment tailored to the underlying etiology [8]. Institutional series also highlight the role of surgery and the importance of evaluating for a causative lesion [9].

Computed tomography may help identify the causative lead point in adult intestinal intussusception, particularly when a homogeneous fat-density lesion is present [10]. Imaging reviews of gastrointestinal lipomas describe typical features that support a benign diagnosis when correlated with clinical and operative findings [11].

We report a case of ileo-ileal intussusception caused by a large pedunculated submucosal lipoma in a young adult male, emphasizing radiologic, intraoperative, and histopathologic correlation.

## Case presentation

A 21-year-old male with no relevant past medical history presented on multiple occasions over a one-month period with episodic abdominal pain, occasionally associated with vomiting. An abdominal ultrasound performed on 02/09/2025 demonstrated focal ileal wall thickening measuring approximately 5-6 cm, which was initially interpreted as ileitis.

On 19/09/2025, the patient re-presented with worsening abdominal pain refractory to analgesic treatment. Physical examination revealed a soft abdomen with mild epigastric tenderness and no signs of peritonitis. Laboratory studies were within normal limits.

**Figure 1:**
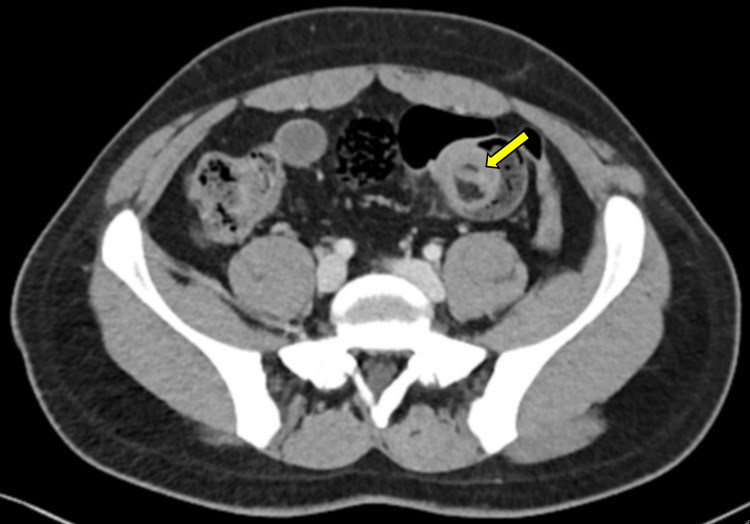
CT imaging of ileo-ileal intussusception. Axial contrast-enhanced CT scan (portal venous phase) demonstrating ileo-ileal intussusception with a central fat-density lesion (arrow, approximately -80 to -120 Hounsfield units), consistent with a submucosal lipoma acting as the lead point. Note the "target sign" with invaginated mesenteric fat and vessels, without evidence of bowel ischemia or perforation.

A contrast-enhanced computed tomography (CT) scan performed on 19/09/2025 demonstrated a long-segment ileo-ileal intussusception involving approximately 15 cm of distal ileum, characterized by a "target sign" measuring approximately 3.5 cm in diameter with invaginated mesenteric fat and vessels, without evidence of bowel ischemia or perforation, as demonstrated by preserved bowel wall enhancement, absence of pneumatosis intestinalis, and absence of free intraperitoneal air (Figure 1). Due to persistent symptoms, a repeat CT scan performed on 21/09/2025 confirmed persistence of the intussusception and identified a well-circumscribed intramural lesion with homogeneous fat attenuation (approximately -80 to -120 Hounsfield units) measuring approximately 23 × 40 × 20 mm at the distal aspect of the intussuscepted segment, consistent with a submucosal lipoma acting as the lead point (Figure 1). Compared to the initial imaging, the repeat CT scan allowed clearer identification of a fat-density intraluminal lesion acting as the lead point, correlating with the progression of clinical symptoms.

**Figure 2:**
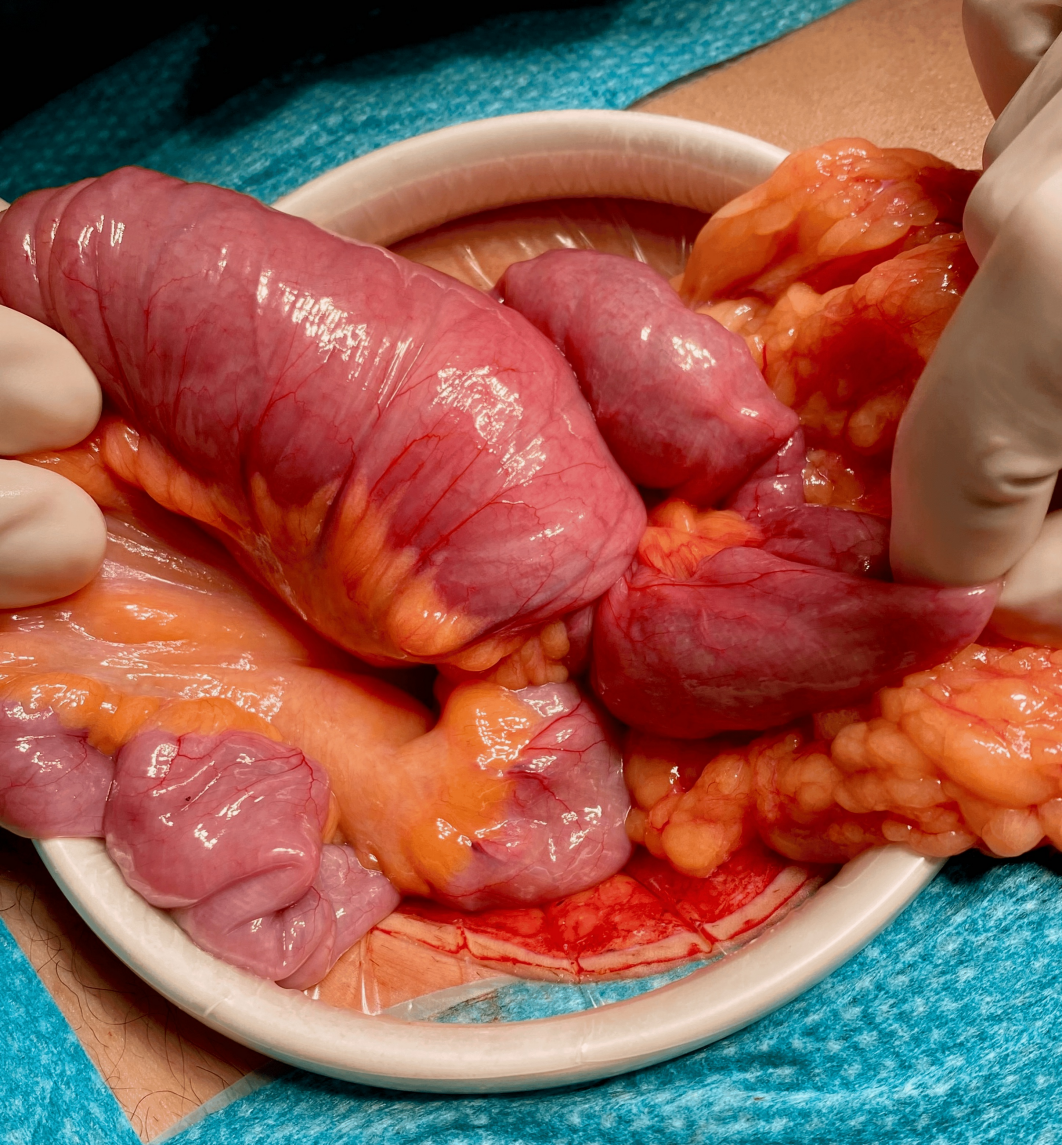
Intraoperative view of ileo-ileal intussusception. Intraoperative photograph showing a long-segment ileo-ileal intussusception prior to reduction.

The patient underwent urgent exploratory laparotomy on 22/09/2025. Intraoperative findings included a 25 cm ileo-ileal intussusception caused by a large pedunculated intraluminal mass (Figure 2).

Manual reduction of the intussusception was performed (Video 1), followed by resection of a 10 cm segment of small bowel and construction of a side-to-side stapled anastomosis.

**Video 1:** Manual reduction of ileo-ileal intussusception. Intraoperative video demonstrating gentle manual reduction of the ileo-ileal intussusception prior to bowel resection, highlighting the extent of bowel involvement and the mobility of the lesion.

**Figure 3:**
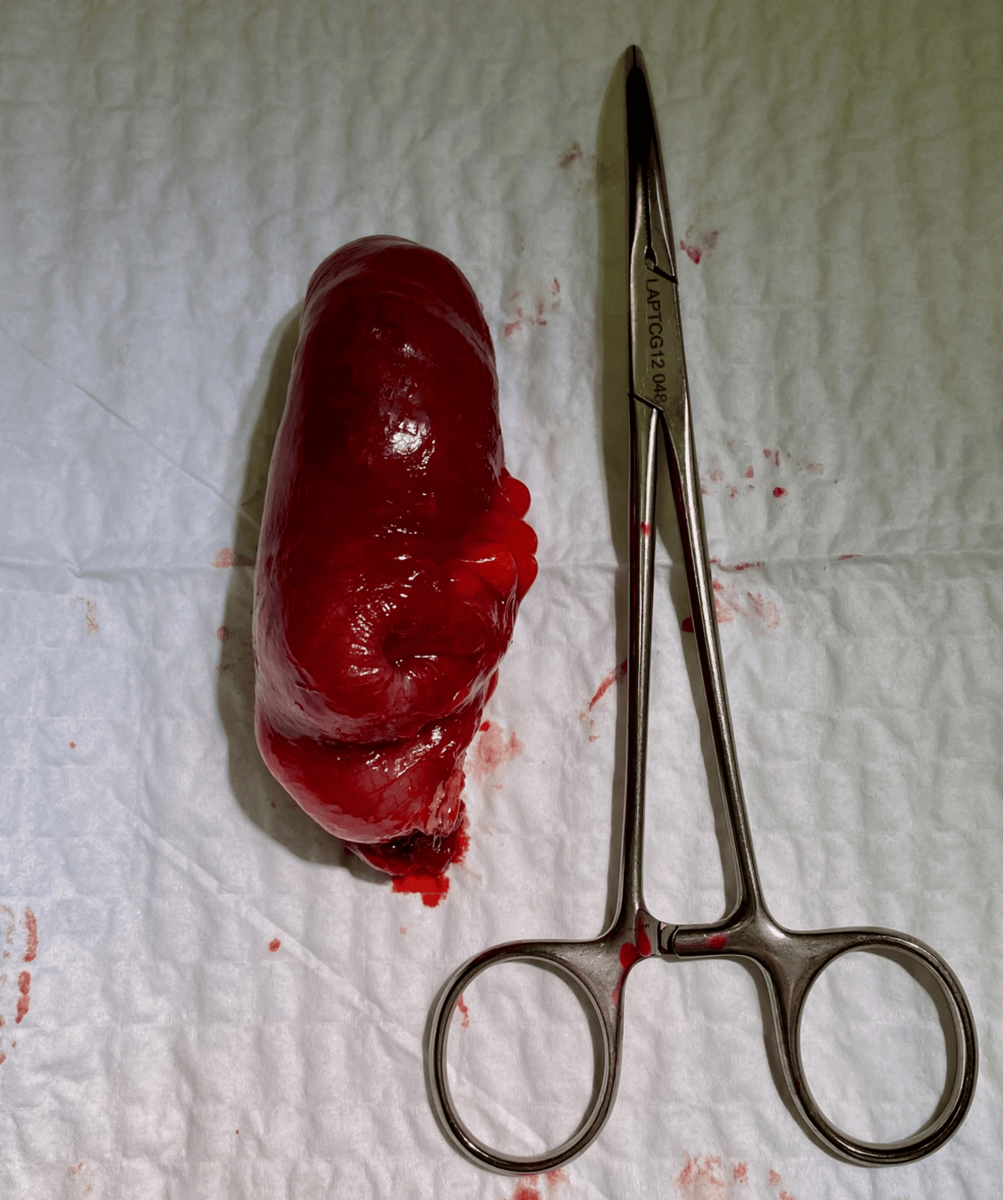
Resected small bowel specimen. Gross specimen of the resected ileal segment containing a large intraluminal pedunculated mass prior to opening.

**Figure 4:**
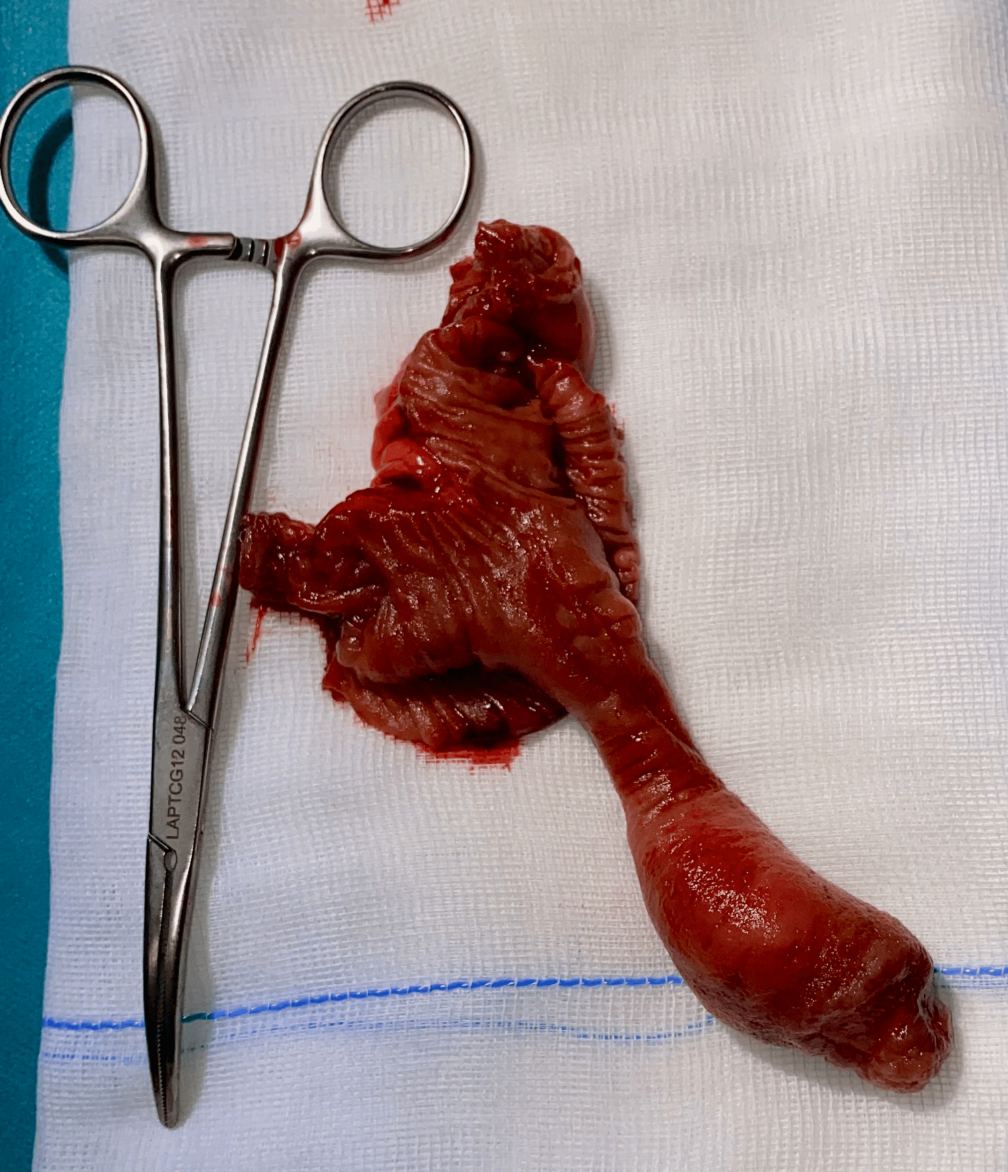
Opened small bowel specimen revealing submucosal lipoma. Opened specimen demonstrating a large pedunculated submucosal lipoma arising from the submucosa, composed of mature adipose tissue, with focal ulceration visible at the distal tip.

**Figure 5:**
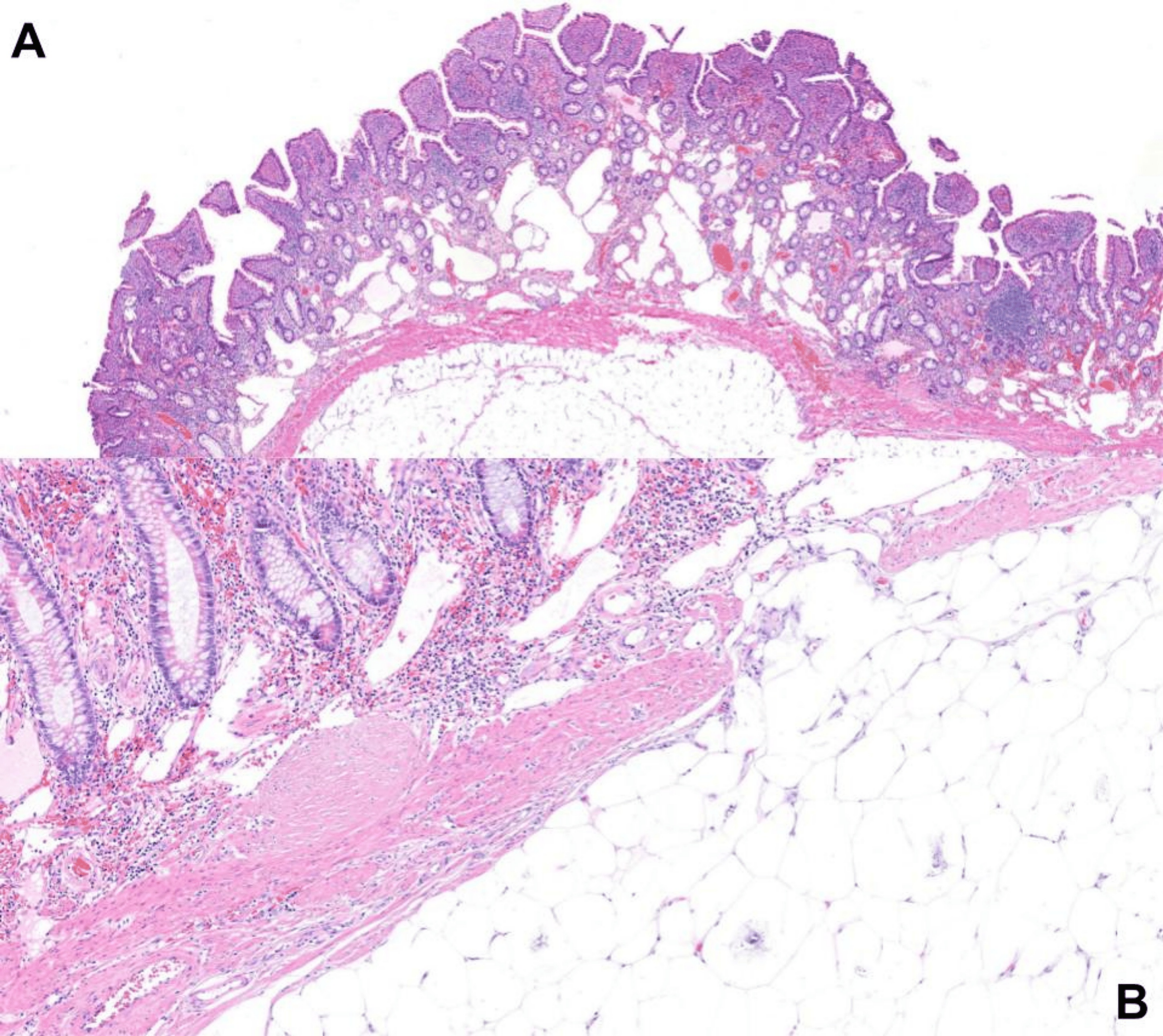
Histopathological examination of the resected lesion. (A) Low-power view showing a submucosal lesion composed of mature adipose tissue underlying the intestinal mucosa. (B) Higher magnification demonstrating mature adipocytes without cytologic atypia, covered by intestinal mucosa with focal ulceration and associated inflammatory infiltrate. No evidence of lipoblasts, necrosis, or malignant features is identified.

Gross inspection of the opened specimen revealed a pedunculated intraluminal lesion measuring approximately 10 cm, with focal ulceration at its distal tip (Figures 3, 4). Histopathological examination demonstrated a well-circumscribed submucosal lesion composed of mature adipose tissue, consistent with a lipoma (Figure 5). The lesion was covered by intestinal mucosa, with focal ulceration at the distal tip showing denuded epithelium, acute inflammatory infiltrate, and fibrinous exudate, consistent with secondary mechanical erosion rather than aggressive behavior. No cytologic atypia, lipoblasts, increased mitotic activity, or necrosis were identified. In the absence of cytologic atypia, lipoblasts, nuclear hyperchromasia, or increased mitotic activity, the morphological features were sufficient to confirm a benign lipoma and exclude atypical lipomatous tumor/well-differentiated liposarcoma. Immunohistochemical studies for MDM2 and CDK4 were therefore not considered necessary, as the histological features did not meet the threshold of suspicion warranting their use. Resection margins were free of lesion.

The postoperative course was uneventful, and the patient was discharged on postoperative day six.

## Discussion

Adult intussusception differs fundamentally from pediatric cases in both etiology and management. In adults, an underlying organic lead point is identified in the majority of cases, most commonly a benign or malignant neoplasm, and surgical management is generally recommended [1,2].

The main surgical differentials for a pedunculated submucosal small bowel mass include gastrointestinal stromal tumor (GIST), neuroendocrine tumor, adenomatous polyp, hamartoma (e.g., in Peutz-Jeghers syndrome), and other mesenchymal tumors such as leiomyoma. Computed tomography reliably demonstrates characteristic findings such as the "target" or "sausage" sign, and plays a central role in identifying the underlying etiology [3].

Adult intussusception has been described in the literature since the mid-20th century, with subsequent large series confirming its consistent association with neoplastic lead points, both benign and malignant [4,5]. More recent systematic reviews and meta-analyses have further confirmed its rarity and the importance of surgical management in the vast majority of cases [6].

Small bowel lipomas represent a rare cause of adult intussusception. While benign, they may cause obstruction, bleeding, or intussusception when they reach sufficient size or develop a pedunculated configuration, and their presentation may mimic malignant pathology, often necessitating surgical exploration. Retrospective institutional series reinforce that surgical resection remains the mainstay of treatment, allowing both definitive management and pathological diagnosis [7,8,9].

Histopathological examination is essential to confirm the benign nature of the lesion and exclude potential mimics. Key differential diagnoses include angiolipoma, spindle cell or pleomorphic lipoma, and most importantly, atypical lipomatous tumor/well-differentiated liposarcoma, which demonstrates cytologic atypia and lipoblasts [11]. The absence of these features in the present case confirms the diagnosis of a benign lipoma. Histopathological evaluation remains the definitive diagnostic tool, allowing differentiation between benign and malignant adipocytic tumors and guiding appropriate management.

Although most gastrointestinal lipomas are sporadic, their occurrence has been loosely associated with conditions such as familial adenomatous polyposis and Cowden syndrome, highlighting the importance of a thorough clinical assessment, though no such syndromic features were present in this patient [11].

Computed tomography is the cornerstone of preoperative diagnosis, with high specificity (approximately 90-95%) for lipomas when a homogeneous fat-attenuation lesion (-50 to -150 Hounsfield units) is identified. Contrast-enhanced CT performed in the portal venous phase allows accurate assessment of bowel wall enhancement, vascular compromise, and identification of fat-attenuation lesions. However, CT sensitivity may be reduced for small lesions, those obscured by bowel wall edema, or cases where telescoping bowel loops partially conceal the lead point. Notably, CT may underestimate the true size of the lesion in cases of pedunculated lipomas, as bowel telescoping may obscure part of the mass within the intussuscepted segment; this phenomenon was consistent with our findings, in which the CT-estimated lesion size (23 × 40 × 20 mm) was smaller than the 10 cm pedunculated mass confirmed at surgery [10,11].

Endoscopic biopsy has a limited role in these lesions due to their submucosal location and the risk of bleeding or ulceration, and is often not feasible in the setting of obstruction. When performed, it may provide tissue diagnosis in accessible lesions, but sensitivity is limited for deep submucosal masses, and the clinical urgency of intussusception frequently precludes its use [10].

This case highlights the importance of multidisciplinary correlation between radiology, surgery, and histopathology in guiding appropriate management and illustrates how imaging findings and intraoperative assessment correlate with previously reported characteristics of lipoma-induced intussusception.

## Conclusions

Adult intussusception should prompt careful evaluation for an underlying structural lesion, as a pathological lead point is frequently present in the majority of cases. Computed tomography plays a central role in diagnosis, demonstrating characteristic imaging findings and allowing preoperative identification of benign causes such as lipomas through recognition of homogeneous fat-attenuation lesions, while also noting that CT may underestimate lesion size in cases of pedunculated tumors due to bowel telescoping. Although benign, large pedunculated submucosal lipomas can result in significant morbidity and usually require surgical management. Histopathological examination is the definitive tool for confirming the benign nature of the lesion and excluding malignant mimics such as well-differentiated liposarcoma. This case highlights the importance of multidisciplinary correlation between radiology, surgery, and histopathology in guiding appropriate diagnosis and treatment in adult intussusception.
